# First record of Picobiinae mites (Acariformes: Syringophilidae) parasitising potoos (Aves: Nyctibiiformes), with description of a new species

**DOI:** 10.1007/s11230-026-10274-y

**Published:** 2026-04-15

**Authors:** Maciej Skoracki, Milena Patan, Till Töpfer, Martin Hromada, Bozena Sikora

**Affiliations:** 1 Department of Animal Morphology, Faculty of Biology, Adam Mickiewicz University, Poznań, Poland; 2 Doctoral School of Natural Sciences, Adam Mickiewicz University, Poznań, Poland; 3 Section Ornithology, Leibniz Institute for the Analysis of Biodiversity Change, Museum Koenig Bonn, Bonn, Germany; 4 Laboratory and Museum of Evolutionary Ecology, Department of Ecology, Faculty of Humanities and Natural Sciences, University of Prešov, Prešov, Slovakia

## Abstract

Quill mites of the subfamily Picobiinae (Acariformes: Syringophilidae) are permanent ectoparasites living within the quills of feathers. Currently, the genus *Gunabopicobia* comprises eight described species that primarily infest pigeons and doves (Columbiformes), except for one species reported from birds-of-paradise (Passeriformes). In this study, we present the first record of *Gunabopicobia* parasitising potoos (Nyctibiiformes). Specimens of *G. nyctibiae* Skoracki & Patan **n. sp.** were collected from the Long-tailed Potoo *Nyctibius aethereus* in Brazil and the Common Potoo *Nyctibius griseus* in Colombia and Paraguay. The finding indicates an unusual host shift between phylogenetically distant avian clades. Moreover, the new species was found inhabiting the quills of small wing coverts, an atypical microhabitat within the Picobiinae subfamily, further suggesting the ecological flexibility of the *Gunabopicobia* lineage. This discovery has important implications for understanding host associations and ecological diversification within Picobiinae and highlights the need to re-evaluate host specificity patterns in the genus *Gunabopicobia*.

## Introduction

Quill mites of the subfamily Picobiinae constitute a morphologically derived and ecologically specialised group within the family Syringophilidae (Acariformes: Prostigmata: Cheyletoidea). These medium- to large-sized mites are permanent and obligatory ectoparasites that inhabit the internal cavities of feather quills, where they feed, reproduce, and complete their entire life cycle (Kethley, 1970, 1971). Over the past two decades, extensive taxonomic revisions (Skoracki, 2011; Skoracki et al., 2016) and large-scale surveys of museum bird collections have substantially expanded our understanding of the diversity and host associations within Picobiinae. Currently, this subfamily comprises 119 valid species, grouped into 18 genera that parasitise approximately 300 bird species from 12 avian orders (Zmudzinski et al., 2023). Despite their worldwide distribution across all zoogeographical regions except Antarctica, most picobiine mites display narrow host ranges, with the majority being mono- or oligoxenous (Skoracki et al., 2016). This high degree of specificity suggests long-term co-diversification and strong phylogenetic constraints between picobiine lineages and their avian hosts. Characteristic, often host-order-specific assemblages of Picobiinae have evolved in parallel with particular avian clades, each typically represented by distinctive genera; for instance, *Lawrencipicobia* Skoracki & Hromada, 2013 is associated with parrots (Psittaciformes), *Charadriineopicobia* Skoracki, Spicer & OConnor, 2014 with shorebirds (Charadriiformes), and *Columbiphilus* Kivganov & Sharafat, 1995 with landfowl (Galliformes) (Skoracki et al., 2016).

The order Nyctibiiformes (potoos) comprises a small group of forest-dwelling, night-active, insectivorous birds restricted to the Neotropics. They are currently considered to represent a single family (Nyctibiidae) with only seven species in two genera, *Phyllaemulor* and *Nyctibius* (AviList Core Team 2025). These cryptically plumaged birds are highly specialised for nocturnal life, with large eyes, wide gapes, and exceptional camouflage allowing them to mimic tree stumps during the day (Winkler et al., 2024). The Nyctibiidae were historically classified within an expanded Caprimulgiformes, together with Caprimulgidae, Podargidae, Steatornithidae, and Aegothelidae, a grouping that reflected the well-supported monophyly of these lineages and underscored their remarkable evolutionary relationships linking the nightbirds with swifts and hummingbirds (Apodiformes) (Cracraft, 2013; del Hoyo & Collar, 2014). However, recent studies have shown that each of these nocturnal bird families represents a distinct order, ranking the Nyctibiidae as an order Nyctibiiformes, consistent with the deep evolutionary divergences estimated among these groups (Chesser et al., 2016; Gill et al., 2023; Clements et al., 2024; Remsen et al., 2025). Despite their wide distribution, the quill mite fauna of potoos remains unexplored, and no representatives of the Picobiinae have been recorded from this avian lineage to date.

In the present study, we report the first record of picobiine mites parasitising birds of the order Nyctibiiformes, represented by the description of a new species, *Gunabopicobia nyctibiae* **n. sp.**, collected from the Long-tailed Potoo *Nyctibius aethereus* (Wied-Neuwied) in Brazil and the Common Potoo *Nyctibius griseus* (Gmelin) in Paraguay and Colombia. This discovery extends the known host range of the genus *Gunabopicobia* beyond Columbiformes, shedding new light on the evolutionary history, host specificity, and potential host-switching events within the Picobiinae.

## Materials and methods

Mites examined in this study were collected from dry bird skins deposited in the two German ornithological collections: the Bavarian Natural History Collections (Staatliche Naturwissenschaftliche Sammlungen Bayerns) in Munich and the Leibniz Institute for the Analysis of Biodiversity Change, Museum Koenig (Leibniz-Institut zur Analyse des Biodiversitätswandels, Museum Koenig) in Bonn. For each host specimen, approximately 5–6 contour feathers from the cloacal region, one small wing covert, and 2–3 under-tail coverts were examined under a ZEISS Discovery.V12 stereomicroscope. Infested feathers were carefully opened with fine-tipped tweezers, and mites were removed and placed in Nesbitt’s solution for three days at room temperature, following the procedure described by Skoracki (2011). Subsequently, the specimens were transferred to 70% ethanol for approximately 30 minutes and then permanently mounted on microscope slides in Hoyer’s medium (Walter & Krantz, 2009). Mounted specimens were examined with a ZEISS Axioscope light microscope (Oberkochen, Germany) equipped with differential interference contrast (DIC) illumination and a camera lucida drawing attachment. In the description, all measurements are given in micrometres (µm). For each character, the measurement of the holotype is followed by the range observed among paratypes in parentheses. The terminology for idiosomal setation follows Grandjean (1939), as adapted for Prostigmata by Kethley (1990). Leg chaetotaxy follows Grandjean (1944), and general morphological terminology follows Kethley (1970), Skoracki (2011), and Skoracki et al. (2016). The scientific and common names of bird hosts are provided according to Clements et al. (2025).

Specimen depositories are abbreviated as follows:

AMU—Adam Mickiewicz University, Department of Animal Morphology, Poznań, Poland.

SNSB-ZSM—Bavarian Natural History Collections, Section Arthropoda Varia, Munich, Germany.

ZFMK—Leibniz Institute for the Analysis of Biodiversity Change, Museum Koenig Bonn, Germany.

**Family Syringophilidae Lavoipierre, 1953**

**Subfamily Picobiinae Johnston & Kethley, 1973**

**Genus:** ***Gunabopicobia*** **Skoracki & Hromada, 2013**

***Gunabopicobia nyctibiae*** Skoracki & Patan n. sp.

*Type-host*: *Nyctibius aethereus* (Wied-Neuwied) (Nyctibiiformes: Nyctibiidae), the Long-tailed Potoo.

*Type-locality*: Brazil: no other data.

*Type-material*: Female holotype and 6 female paratypes (reg. no. AMU-MS 22-0202-087); mites collected by M. Skoracki & B. Sikora; host specimen collected by H. Leucht and deposited in the SNSB-ZSM (uncatalogued).

*Site in host*: Quill of small wing covert.

*Type material deposition*: Female holotype and 2 female paratypes are deposited in the AMU, 4 female paratypes in the SNSB-ZSM.

*Additional-material*: Ex. quill of small wing covert of the Common Potoo *Nyctibius griseus* (Gmelin) (Nyctibiiformes: Nyctibiidae) (host reg. no. ZFMK_ORN 1957.1688, female); Paraguay: 50 km east of Orloff, Fernheim Colony, Boqueron Department, 31 March 1957, leg. J. Unger—2 females deposited in the ZFMK and 1 female in the AMU (reg. no. AMU-MS 25-0623-010). Ex. same host species and habitat (host in the ZSM, uncatalogued); Colombia: Cauca Department, Popayán, 11 May 1964, leg. Orozco—2 females deposited in the AMU (reg. no. AMU-MS 25-0907-001).

*ZooBank registration*: The Life Science Identifier (LSID) for *Gunabopicobia nyctibiae* **n. sp.** is urn:lsid:zoobank.org:act: xxxx.

*Etymology*: The specific name is taken from the generic name of the host, *Nyctibius*.

**Fig. 1:**
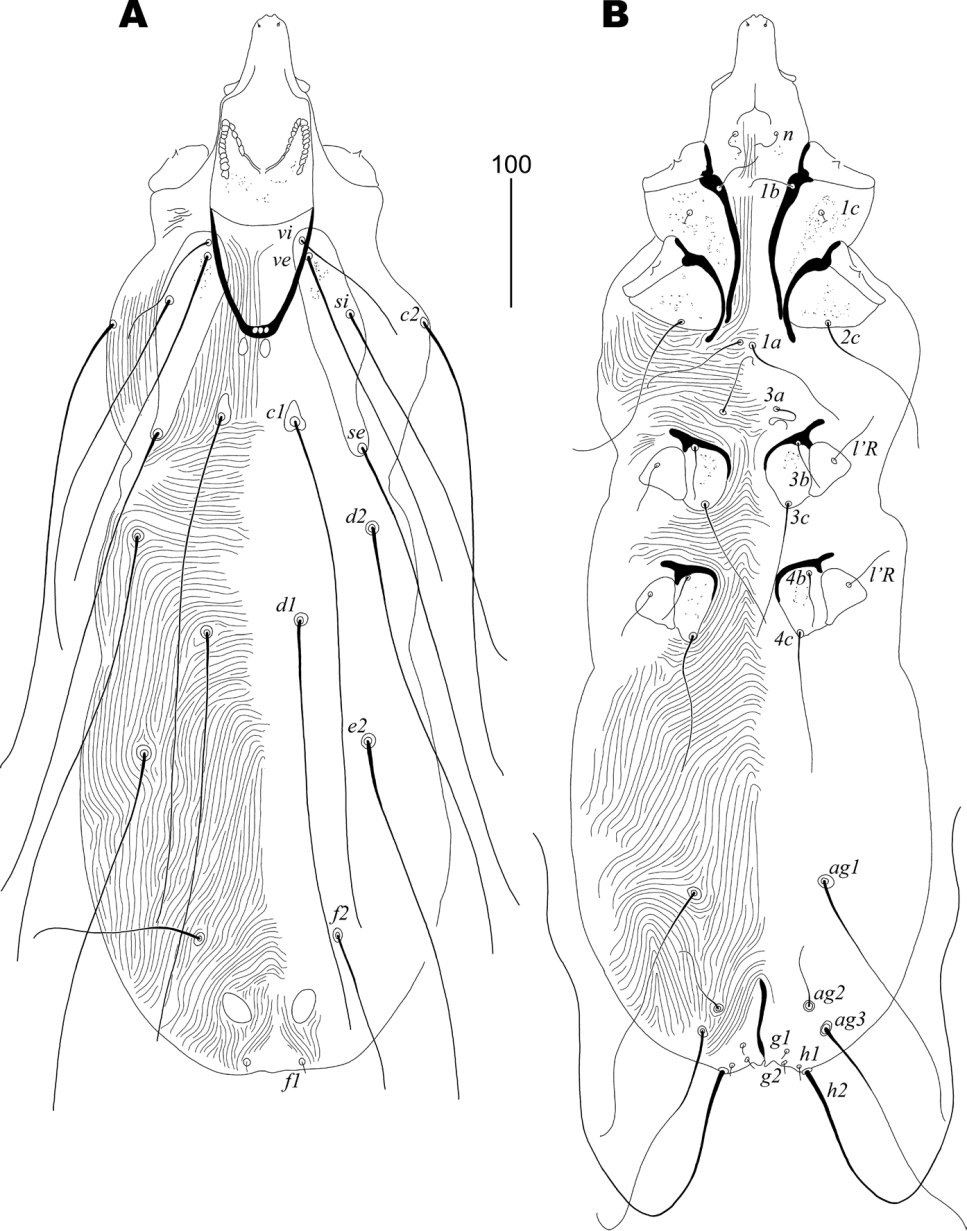
*Gunabopicobia nyctibiae* Skoracki & Patan **n. sp.**, female. **A** Dorsal view; **B** Ventral view. *Scale-bar*: 100 µm

**Fig. 2:**
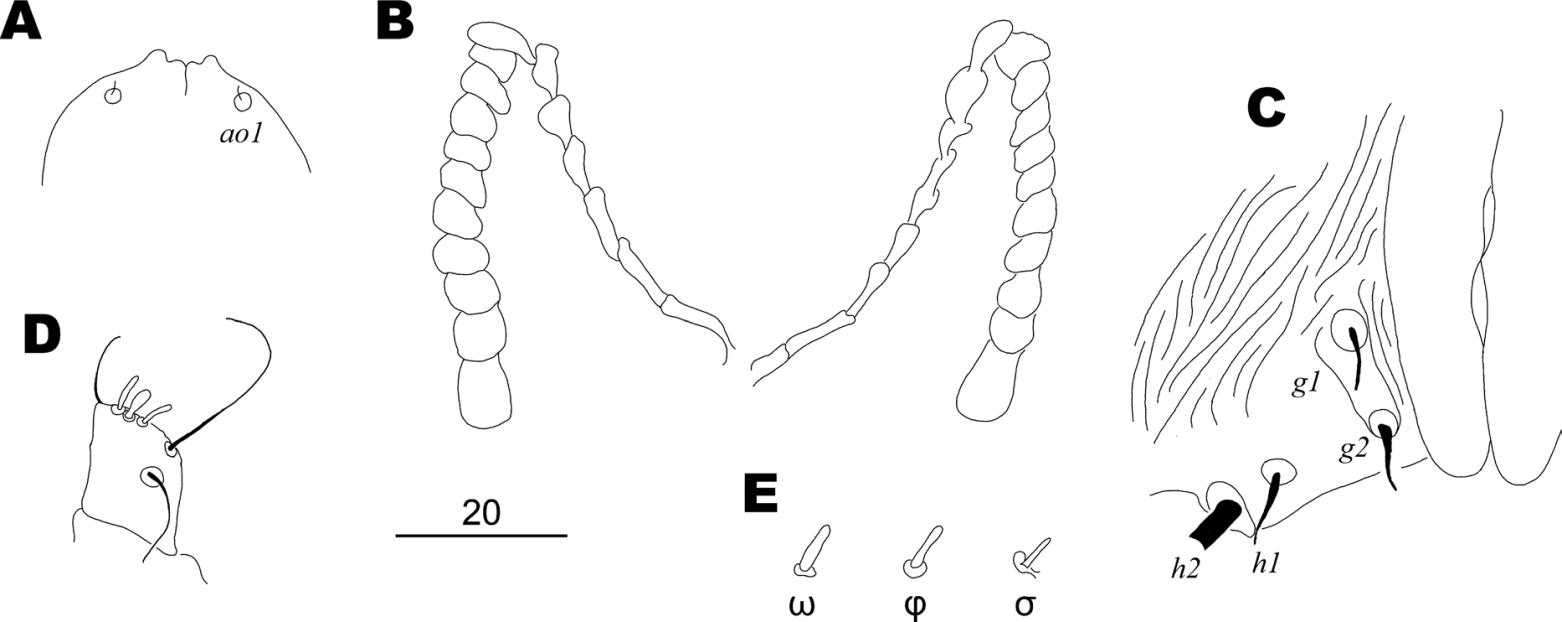
*Gunabopicobia nyctibiae* Skoracki & Patan **n. sp.**, female. **A** Hypostomal apex in dorsal view; **B** Peritremes; **C** Genital region; **D** Tibiotarsus of palp in ventral view; **E** solenidia of leg I. *Scale-bar*: 20 µm

Description (Figs. 1 and 2)

Female: Total body length 880 in holotype (820–875 in six female paratypes). *Gnathosoma*. Hypostomal apex with pair of small blunt-ended protuberances. Infracapitulum sparsely punctate. Stylophore 250 (250–270) long, exposed portion of stylophore punctate, 160 (160–165) long. Movable cheliceral digit 210 (200–215) long, edentate on proximal end. Each medial branch of peritremes has 7–10 chambers, each lateral branch has ten chambers. *Idiosoma*. Propodonotal shield divided into two longitudinal sclerites bearing bases of setae *vi*, *ve*, *si*, and *se*, punctate between bases of setae *ve* and *si*; additionally, two small apunctate sclerites in the middle of propodonotum present. Lengths of propodonotal setae *vi* 115 (110–130), *ve* (270), *si* 290 (255–275), *se* 375 (370–385), *c1* 380 (380–400), *c2* 395 (355–360). Lengths between setal bases: *vi*-*vi* 70 (70–75), *ve*-*ve* 75 (75–80), *si*-*si* 140 (135–140), *se*-*se* 160 (160–165), *c1*-*c1* 60 (55–60), *vi*-*si* 70 (70–75), *vi*-*se* 160 (155–170), *vi*-*c1* 145 (140–145). Hysteronotal shield absent. Pygidial shield reduced to two small oval and apunctate sclerites. Lengths of hysteronotal setae: *d1* 335 (320–345), *d2* 370 (350–365), *e2* 300 (275–290), *f1* 10 (8–10), *f2* 130 (130–160), *h1* 10 (8–10), *h2* (460–480). Lengths between setal bases: *f1*-*f2* 90 (90–100), *f2*-*f2* 110 (110–115). Both pairs of genital setae (*g1* and *g2*) equal in length. Aggenital setae *ag1* and *ag3* about four times longer than *ag2*; bases of setae *ag2* situated closer to *ag3* than to *ag1*, lengths between setal bases *ag1*-*ag2* 90 (90–100), *ag2*-*ag3* 20 (20–25). All coxal fields punctate. Lengths of ventral setae: *g1* and *g2* 10 (8–10), *ag1* (205–210), *ag2* 45 (45–50), *ag3* (180–200), *3b* 40 (35–40), *4b* 40 (35–40), *3c* 110 (100–110), *4c* 110 (105–125).

Male: Unknown.

### Differential diagnosis

*Gunabopicobia nyctibiae* **n. sp.** is morphologically similar to *G. metropelia* Kaszewska, Skoracki & Hromada, 2018, described from *Metriopelia melanoptera* (Molina) (Columbiformes: Columbidae) in Argentina (Kaszewska et al., 2018) by the presence of separated bases of setae *1a*–*1a*, the punctate infracapitulum, and the punctate stylophoral shield; the bases of setae *c1* are situated anterior to the level of setal bases *se*. This new species differs from *G. metropelia* in the following features: in females of *G. nyctibiae*, each lateral branch of the peritremes has 10 chambers; the propodonotal shield is divided into two longitudinal and two small median sclerites; lengths of propodonotal setae *vi*, *ve*, and *si* are 110–130, 270, and 255–290 µm, respectively; the bases of setae *f2* are situated far anterior to the level of setae *f1*, and setae *f1* are about 10 µm long. In females of *G. metropelia*, each lateral branch of the peritremes has 5–6 chambers; the propodonotal shield is entire; lengths of propodonotal setae *vi*, *ve*, and *si* are 10, 10–15, and 20 µm, respectively; the bases of setae *f2* are situated close together and slightly anterior to the level of setae *f1*, and setae *f1* are 80–90 µm long.

## Discussion

Until now, the genus *Gunabopicobia* comprised eight described species, seven of which parasitise on 24 species of pigeons and doves (Columbiformes: Columbidae) belonging to the genera *Caloenas*, *Claravis*, *Columba*, *Ducula*, *Geotrygon*, *Leptotila*, *Leucosarcia*, *Metriopelia*, *Patagioenas*, *Ptilinopus*, *Spilopelia*, *Streptopelia*, *Zenaida*, and *Zentrygon* (Kaszewska et al., 2018; Kaszewska-Gilas et al., 2021). This distribution pattern highlights the strong host specificity of *Gunabopicobia* mites toward columbiform birds. One species, *G. garylarsoni*, has been recorded from two species of birds-of-paradise of the genera *Paradisaea* and *Seleucidis* (Passeriformes: Paradisaeidae) (Sikora et al., 2023). The occurrence of *G. garylarsoni* Sikora et al., 2023 on the birds-of-paradise, a host group phylogenetically distant from the columbiform hosts, was interpreted as the result of a host-switching event from pigeons to passerines, possibly facilitated by ecological or behavioural contact between these birds rather than by long-term coevolutionary processes (Sikora et al., 2023).

The discovery of *G. nyctibiae* **n. sp.** on the Long-tailed Potoo and the Common Potoo represents a remarkable example of a host shift. Phylogenetically, Nyctibiiformes belong to the Strisores, a clade that also includes Aegotheliformes, Apodiformes, Caprimulgiformes, Steatornithiformes, and Podargiformes, which are only distantly related to the Columbiformes (Jarvis et al., 2014; Prum et al., 2015; Chen et al., 2019; Chen & Field, 2020; Stiller et al., 2024). This finding thus indicates a substantial host transition across deep evolutionary boundaries. It suggests that the ancestral lineage of *Gunabopicobia* may possess a degree of ecological plasticity allowing it to colonise unrelated avian taxa under particular ecological conditions. The high degree of ecological flexibility is further supported by the fact that *G. nyctibiae* **n. sp.** was collected from the quills of small wing coverts, whereas all other known picobiine species have been recorded exclusively from body feather quills, except for *Calamincola lobatus* (Casto, 1977), found in the quills of secondary remiges, and *Lawrencipicobia poicephali* Skoracki & Dabert, 2002, inhabiting the quill wall of upper and undertail coverts (Casto, 1977; Skoracki et al., 2019).

Host-switching in quill mites is generally regarded as a rare phenomenon due to the highly specialised microhabitat they occupy and their close adaptation to host feather morphology. However, occasional host transfers (horizontal transfer) have been documented in a few cases of Syringophilidae-host interactions, in which quill mites can move between phylogenetically unrelated host groups. Notable examples include transfers between predator and prey species (Nattress, 2011) or among hosts sharing similar ecological niches, particularly in gregarious or colony-nesting birds (Skoracki et al., 2021). In addition, contact during copulation has been proposed as another potential pathway for horizontal transmission in quill mites parasitising brood-parasitic cuckoos or birds-of-paradise (Hromada et al., 2016; Sikora et al., 2023). In the case of *G. nyctibiae* **n. sp.**, the mechanism behind its occurrence on the usually solitary nyctibiiform hosts remains uncertain. It may represent an ancient colonisation event, followed by subsequent radiation within Nyctibiiformes, or, alternatively, an isolated instance of cross-infestation from a columbiform host sharing similar ecological niches in tropical forests of South America. This finding highlights the importance of comprehensive examinations of the preserved avian specimen material to uncover hidden diversity and trace unexpected evolutionary connections between mites and their hosts. Further surveys across the Strisores clade are needed to clarify whether *G. nyctibiae* represents an isolated case of host colonisation or the first member of a broader, yet undescribed, radiation of *Gunabopicobia* mites associated with birds of the order Nyctibiiformes or the whole Strisores.

## Data Availability

No datasets were generated or analysed during the current study.
